# Mobilization with movement is effective in patients operated for distal radius fracture

**DOI:** 10.1590/1806-9282.20250577

**Published:** 2025-09-19

**Authors:** Pu Wan, Zhi Li

**Affiliations:** 1 Dalian Medical University, The First Affiliated Hospital, Department of Joint and Sports Medicine – Dalian, China.

Dear Editor,

We read with interest the recent randomized controlled trial by Horoz et al.^1^ evaluating Mulligan's mobilization with movement (MWM) for rehabilitation of distal radius fractures (DRFs). The study revealed MWM's efficacy in pain-free functional recovery among postoperative DRF patients. In this trial, 53 patients were randomized into two groups, with the experimental group receiving MWM in addition to the standard rehabilitation program used in the control group. Through measurements of volar tilt, radial inclination, grip strength, and wrist assessment questionnaires, the study demonstrated MWM's significant improvements in grip strength and pronation function for postoperative DRF patients. While this research provides valuable insights into post-DRF recovery, we wish to highlight several critical points to refine the interpretation of its findings.

First, MWM's core mechanism involves correcting articular malalignment to improve biomechanical function, primarily applied in musculoskeletal disorders to relieve pain and restore joint mobility^2^. Although current clinical data support its broad effectiveness, the study's differential efficacy across fracture types warrants deeper exploration. Compared with stable fractures, comminuted fractures typically entail higher complication risks and poorer prognoses due to severe anatomical disruption. Conducting subgroup analyses could more precisely evaluate the universal applicability of MWM across various fracture types. For instance, fractures preserving articular surface integrity might demonstrate better responsiveness to early mobilization therapies, given their relatively intact anatomical continuity, thereby achieving greater clinical benefits. This perspective could elucidate the observed heterogeneity in MWM's clinical benefits across subgroups.

Furthermore, while the study shows that MWM significantly enhanced forearm pronation and grip strength, its underlying mechanisms merit further investigation. The observed pain relief might stem from MWM-induced mechanical hypoalgesia via endogenous pain modulation^3^, potentially linked to grip strength improvement. The mobility restoration could relate to effective dorsal capsular release, a hypothesis requiring verification through direct imaging modalities such as magnetic resonance imaging (MRI) or ultrasound to visualize capsular changes. Such mechanistic exploration would strengthen the theoretical foundation for MWM's clinical application.

In conclusion, we commend the authors’ rigorous methodology aligning with enhanced recovery after surgery (ERAS) principles, providing valuable guidance for DRF management. Future studies building upon this work should address the raised considerations to optimize personalized rehabilitation protocols.

## Data Availability

The datasets generated and/or analyzed during the current study are available from the corresponding author upon reasonable request.
